# Diversity in the Enteric Viruses Detected in Outbreaks of Gastroenteritis from Mumbai, Western India

**DOI:** 10.3390/ijerph9030895

**Published:** 2012-03-14

**Authors:** Shobha Chitambar, Varanasi Gopalkrishna, Preeti Chhabra, Pooja Patil, Harsha Verma, Anismrita Lahon, Ritu Arora, Vaishali Tatte, Sujata Ranshing, Ganesh Dhale, Rajendra Kolhapure, Sanjay Tikute, Jagannath Kulkarni, Renu Bhardwaj, Sulbha Akarte, Sashikant Pawar

**Affiliations:** 1 Enteric Viruses Group, National Institute of Virology, 20-A, Dr. Ambedkar Road, Pune 411001, India; Email: gopalvk58@hotmail.com (V.G.); preetischhabra@gmail.com (P.C.); patilpooja15@hotmail.com (P.P.); harshavverma@gmail.com (H.V.); ritusarora@gmail.com (R.A.); anismritalahon@yahoo.in (A.L.); vprabhavale@yahoo.com (V.T.); sujata_ranshing@yahoo.com (S.R.); g_dhale@yahoo.com (G.D.); nivrota@hotmail.com (R.K.); sanjaytikute@yahoo.co.in (S.T.); 2 Department of Pathology, Gokuldas Tejpal Hospital, Lokmanya Tilak Marg, Fort, Mumbai 400001, India; Email: drjmkulkarni@gmail.com; 3 Department of Microbiology, Sir Jamshedjee Jeejeebhoy Hospital, Byculla, Mumbai 400008, India; Email: renu.bharadwaj@gmail.com; 4 Department of Preventive and Social Medicine, Sir Jamshedjee Jeejeebhoy Hospital, Byculla, Mumbai 400008, India; Email: svakarte@hotmail.com (S.A.); nivrota@yahoo.com (S.P.)

**Keywords:** adenovirus, Aichivirus, astrovirus, enterovirus, norovirus, rotavirus, gastroenteritis outbreak

## Abstract

Faecal specimens collected from two outbreaks of acute gastroenteritis that occurred in southern Mumbai, India in March and October, 2006 were tested for seven different enteric viruses. Among the 218 specimens tested, 95 (43.6%) were positive, 73 (76.8%) for a single virus and 22 (23.2%) for multiple viruses. Single viral infections in both, March and October showed predominance of enterovirus (EV, 33.3% and 40%) and rotavirus A (RVA, 33.3% and 25%). The other viruses detected in these months were norovirus (NoV, 12.1% and 10%), rotavirus B (RVB, 12.1% and 10%), enteric adenovirus (AdV, 6.1% and 7.5%), Aichivirus (AiV, 3% and 7.5%) and human astrovirus (HAstV, 3% and 0%). Mixed viral infections were largely represented by two viruses (84.6% and 88.9%), a small proportion showed presence of three (7.7% and 11%) and four (7.7% and 0%) viruses in the two outbreaks. Genotyping of the viruses revealed predominance of RVA G2P[4], RVB G2 (Indian Bangladeshi lineage), NoV GII.4, AdV-40, HAstV-8 and AiV B types. VP1/2A junction region based genotyping showed presence of 11 different serotypes of EVs. Although no virus was detected in the tested water samples, examination of both water and sewage pipelines in gastroenteritis affected localities indicated leakages and possibility of contamination of drinking water with sewage water. Coexistence of multiple enteric viruses during the two outbreaks of gastroenteritis emphasizes the need to expand such investigations to other parts of India.

## 1. Introduction

Acute gastroenteritis is the third leading cause of mortality in the World [1]. The causative agents of gastroenteritis include bacteria, parasites and viruses. The viruses associated with gastroenteritis are rotaviruses (RVs), calciviruses [noroviruses (NoVs) and sapoviruses (SaVs)], enteric adenoviruses (AdVs), human astroviruses (HAstVs), Aichiviruses (AiVs), toroviruses, coronaviruses and picobirnaviruses [2]. Enteroviruses (EVs) have been also found to be associated with acute gastroenteritis [3]. Recently, Sali/Klasseviruses are also enlisted as enteric viral pathogens [4].

RVAs and RVBs, members of the family Reoviridae, carry a double stranded RNA genome, 18.5 kb in size, with 11 gene segments. Based on the characteristics of VP6, the major structural protein, RVAs are divided into seven groups (A-G). RVAs known to infect young humans and animals have been classified into 27 G and 35 P types on the basis of VP7 (G, glycoprotein) and VP4 (P, protease sensitive) proteins, respectively [5]. RVBs are genetically and antigenically distinct from RVAs and have been found to infect humans and different species of animals. RVB infections have been identified in China, Bangladesh, India and Myanmar to date and often found to cause infections in adolescents and adults and occasionally in children [6,7].

NoVs, members of the family Caliciviridae, possess a single-stranded RNA genome of 7.5–7.7 kb and have been classified into five major groups, Genogroup I (GI) to Genogroup V (GV). GI, GII and GIII have been subdivided into eight, 21 and two genetic clusters, respectively, whereas GIV and GV have one cluster each [8]. Enteric AdVs carrying DNA of 33-45 kb belong to the family Adenoviridae. Based on the biological and genetic characteristics, AdVs are classified into six subgenera, A–F that include more than 51 human AdV serotypes. Among these, subgenera ‘F’ carrying AdV types 40 and 41 and “A” carrying AdV types 12, 18 and 31 have been found to be associated with acute gastroenteritis [9,10]. HAstVs belong to the family Astroviridae and carry RNA genome of 6.8–7.6 kb in size. Based on the antigenic reactivity of the capsid proteins, these viruses have been classified into eight serotypes, 1–8 [11]. 

EVs, members of the family Picornaviridae, possess a single stranded positive sense RNA genome of 7.5 kb and have been classified into four species, Human enterovirus (HEV) A,B,C,D with more than 90 serotypes [12]. AiV of the genus Kobuvirus, family Picornaviridae consists of a single-stranded positive-sense RNA molecule of 8.2 kb nucleotides excluding a poly (A) tail. Based on the 3C-3D junction and VP1 regions, the AiV genome has been classified into three genotypes A, B and C [13]. 

In India, sporadic infections of gastroenteritis have been investigated extensively for enteric viruses. Rotaviruses followed by NoVs, enteric AdVs and HAstVs are the most frequently identified causative agents of viral gastroenteritis [10,14,15,16,17,18]. Although occurrence of outbreaks of gastroenteritis has been documented in the records of the State Surveillance Unit, Integrated Disease Surveillance Project, Maharashtra, investigations to unfold viral aetiology have been rarely carried out. In the present study, two outbreaks of acute gastroenteritis that occurred in southern Mumbai, western India in the months of March and October 2006 were investigated for multiple enteric viral agents to determine their contribution in causing gastroenteritis.

## 2. Experimental Section

Mumbai is the capital of Maharashtra state and most populous city in India, with a total population of 18.4 million and a total area of 603.4 km^2^. Apart from one dam six major lakes supply water to the city [19]. Mumbai has a tropical climate, mostly warm and humid in nature. The occurrence of gastroenteritis outbreaks in southern Mumbai was reported in the months of March and October, 2006. 

### 2.1. Epidemiological Investigation

To ascertain the occurrence of outbreaks of gastroenteritis, medical record of all gastroenteritis cases referred to Gokuldas Tejpal Hospital (GTH) and Sir Jamshedjee Jeejeebhoy Hospital (JJH) from January to December, 2006, was examined and the information on age, gender, symptoms, date of onset and admission of patients was acquired. The number of gastroenteritis cases admitted to the pediatric and adult wards of GTH and JJH in the previous year was also recorded. Any inhabitant of the gastroenteritis affected localities, admitted for ≥3 loose or watery stools a day with or without vomiting, fever and abdominal pain at GTH during 13th–26th March (11–12 weeks), 2006 and at JJH during 5th–15th October (40–41 weeks), 2006 was considered an outbreak case. 

### 2.2. Specimens

Faecal specimens were collected from 72 (30 children ≤10 years, 5 adolescents aged 11–17 years and 37 adults ≥18 years) and 146 (61 children ≤10 years, 6 adolescents aged 11–17 years and 79 adults ≥18 years) patients hospitalized at GTH and JJH, respectively, during the outbreak periods. Thirty percent faecal suspensions were prepared in 0.01 M phosphate buffered saline (PBS), pH 7.4. The suspensions were centrifuged at 10,000 rpm for 10 min to remove the debris and supernatants were stored in aliquots at −70 °C until tested. 

### 2.3. Laboratory Investigation

#### 2.3.1. Nucleic Acid Extraction, PCR and RT-PCRs

The viral nucleic acids were extracted from the faecal specimens using guanidium isothiocyanate (Invitrogen, Carlsbad, CA; Imperial, OH, USA) or spin columns (Qiagen, Hilden, Germany) as per manufacturer’s instructions. All specimens were tested by conventional RT-PCR targeting 220 nucleotides of the RVA VP6 gene as described earlier [20]. Genotyping of RVA strains was carried out by multiplex PCR of VP7 and VP4 genes according to the protocols described previously [17,21,22]. 

Newly designed primers NSP2-AF: GCCATCAGACAGAGAATGTGTTGCA (primer positions: 112–136), NSP2-BR: CCAATCAGTCACAAGAGTCCATAGT (primer positions: 340–316) and NSP2-CR: TTGTCTGCCGAAGCTAAAACATCC (primer positions: 432–409) based on CAL-1 strain (AY238383) targeting against 321 bp (primers NSP2-AF and NSP2-CR) and 229 bp (primers NSP2-AF and NSP2-BR) of NSP2 gene were employed to detect the presence of RVB in the faecal specimens by semi-nested RT-PCR assay. PCR conditions involved an initial reverse transcription of 30 min at 50 °C, followed by PCR activation at 95 °C for 15 min and 35 cycles of amplification (1 min at 94 °C, 30 sec at 55 °C and 1 min at 72 °C) with final extension at 72 °C for 7 min.

The presence of NoV GI and GII, AdV, HAstV, EV and AiV RNA in the faecal specimens was detected by amplification of RdRp region A (126 bp), hexon (300 bp), ORF 1a (289 bp), 5′ NCR (404 bp), 3C-3D junction (265 bp) regions, respectively as described earlier [23,24,25,26,27,28]. This was followed by genotyping of NoV, HAstV and EV strains with primers specific for VP1 [region C, 330 (GI)/344 bp (GII)], ORF2 (232 bp), VP1/2A (450 bp) regions, respectively as described previously [18,29,30]. 

#### 2.3.2. Nucleotide Sequencing and Phylogentic Analyses

All of the PCR products were electrophoresed in 2% agarose gel containing ethidium bromide (0.5 µg/mL) and visualized under UV transilluminator. PCR amplicons were excised from the gel for purification (QIAquick, Qiagen, Hilden, Germany) and cycle sequencing was carried out using BigDye^®^ Terminator v3.1 cycle sequencing kit (Applied Biosystems, Foster City, CA, USA). The sequences were collected from automated DNA sequencer ABI 3130XL (Applied Biosystems). 

Sequence identity was determined through BLAST and multiple sequence alignment was carried out with CLUSTAL W program. The phylogenetic analysis of aligned sequences was carried out using MEGA 4 [31]. The phylogenetic tree was generated with neighbor-joining algorithm and Kimura 2-parameter distance model. The reliability of phylogenetic tree was tested by applying bootstrap test with 1000 bootstrap replications.

#### 2.3.3. Nucleotide Sequence Accession Numbers

The nucleotide sequences of the strains examined in the study have been deposited in GenBank under the accession numbers, HQ268736–HQ268770 for RVA, HQ268794–HQ268804 for RVB, EU921357–EU921362 and EU921396–EU921399 for NoV, HQ268775–HQ268790 for AdV, HQ268791–HQ268793 for HAstV, HQ662310–HQ662328 for EV and HQ425480–HQ425483 for AiV.

### 2.4. Environmental Investigation

The hygienic conditions of the areas affected by gastroenteritis outbreaks and water supply and sewage pipelines were inspected by the health officials of Mumbai. A drinking water sample of 5 liters was collected on 17th March, 2006 from each of the two sources utilized by the consumers. Water samples were concentrated from 5 liters to ~200 mL by membrane based ultrafiltration using a membrane with an exclusion limit of 60,000 Daltons [32] and subsequently a volume of 200 mL was concentrated to ~2 mL in an Amicon cell (Millipore) and stored in aliquots at −70 °C till tested for enteric viruses (RVA, RVB, NoV, AdV, HAstV, EV and AiV).

## 3. Results

### 3.1. Epidemiological Investigation

**Figure 1:**
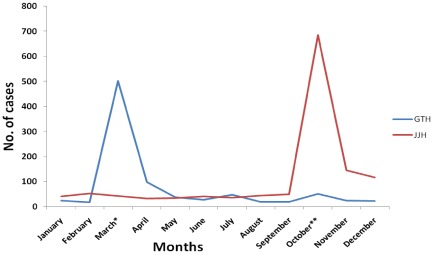
Frequencies of admission of patients with gastroenteritis referred to GTH and JJH in the year 2006. * includes 66 children, 12 adolescents and 222 adults hospitalized during 13th–26th March, 2006; ** includes 53 children, 13 adolescents and 198 adults hospitalized during 5th–15th October, 2006.

Two outbreaks of acute gastroenteritis were reported in the months of March and October 2006, respectively, from two and one localities of southern Mumbai. The outbreak affected localities, each 1–1.5 km^2^ in area that was occupied by 4500 and 7000 inhabitants respectively. Nearly 7% and 4% of the inhabitants were affected by the disease, respectively, during the months of March and October, 2006. In order to establish the incidence of gastroenteritis outbreaks, the number of gastroenteritis cases admitted monthly to GTH and JJH was recorded for the year, 2006. Figure 1 shows that the frequencies of admission for gastroenteritis in the hospitals were increased remarkably in GTH and JJH in March and October, 2006 respectively. The records of both hospitals also showed higher number of admissions in 2006 [886 (GTH), 1322 (JJH)] as compared to those in 2005 [278 (GTH), 477 (JJH)].

During March (weeks 11–12) and October (weeks 40–41) a total of 300 (66 children ≤10 years, 12 adolescents aged 11–17 years and 222 adults ≥18 years) and 264 (53 children aged ≤10 years, 13 adolescents aged 11–17 years and 198 adults ≥18 years) patients with gastroenteritis were admitted to GTH and JJH, respectively. Analysis of clinical features of the patients recorded in both outbreaks indicated presence of diarrhea in 95–97.6%, vomiting in 83.3–86.2% and fever in 24–26.2% of the patients. The majority of the patients suffered from the illness for 2 to 3 days. 

### 3.2. Enteric Virus Positivity

Of the 218 faecal specimens from the two outbreaks of gastroenteritis, 95 (43.6%) tested positive for enteric virus. These included 46 (63.9%) of 72 and 49 (33.6%) of 146 from March and October, 2006 respectively. Table 1, Table 2 show the distribution of single and mixed viruses detected in the two outbreaks Single viral infections of RVAs and EVs predominated in both outbreaks. Other viruses (RVBs, NoVs, AdVs, HAstVs and AiVs) were detected at lower levels (Table 1). The majority (84.6% and 88.9%) of patients with mixed enteric viral infections showed the presence of two viruses in both outbreaks. Mixed infections with three viruses were detected only in 7.7% and 11.1% of the patients. An infant, aged 10 months admitted to GTH in March showed infection with four viruses (Table 2). 

**Table 1:** Distribution of single enteric viruses detected in the outbreak cases of gastroenteritis.

Enteric virus	No. positive (%)	
	March 2006 (*n* = 33)	October 2006 (*n* = 40)
RVA	11 (33.3)	10 (25)
RVB	4 (12.1)	4 (10)
NoV	4 (12.1)	4 (10)
AdV	2 (6.1)	3 (7.5)
HAstV	1 (3)	0
EV	10 (30.3)	16 (40)
AiV	1 (3)	3 (7.5)

**Table 2:** Distribution of mixed enteric viruses detected in the outbreak cases of gastroenteritis.

Period of outbreak		RVA	RVB	NoV	AdV	HAstV	EV
March, 2006 (*n* = 13)	M-689	+		+			
	M-791	+		+			
	M-633	+	+	+	+		
	M-705	+		+			
	M-693	+					+
	M-636	+					+
	M-778	+			+		
	M-651	+			+		
	M-777			+	+		
	M-771				+		+
	M-714		+		+		+
	M-635				+		+
	M-634		+				+
October, 2006 (*n* = 9)	M-836	+					+
	M-859	+					+
	M-029	+					+
	M-038	+			+		
	M-308	+			+		
	M-864	+				+	
	M-857			+			+
	M-361			+	+	+	
	M-364			+	+		

### 3.3. Age Distribution

Enteric virus positivity was not different in children and adults, however was higher as compared to that of adolescents in both outbreaks (45.7% and 50% *vs*. 4.3% in March, 49% and 49% *vs*. 2% in October).

### 3.4. Nucleotide Sequencing and Phylogenetic Analyses

The partial regions of conserved and/or variable genes of all of the enteric viruses detected in single and mixed infections were sequenced and analyzed phylogenetically. 

#### 3.4.1. RVA

**Figure 2:**
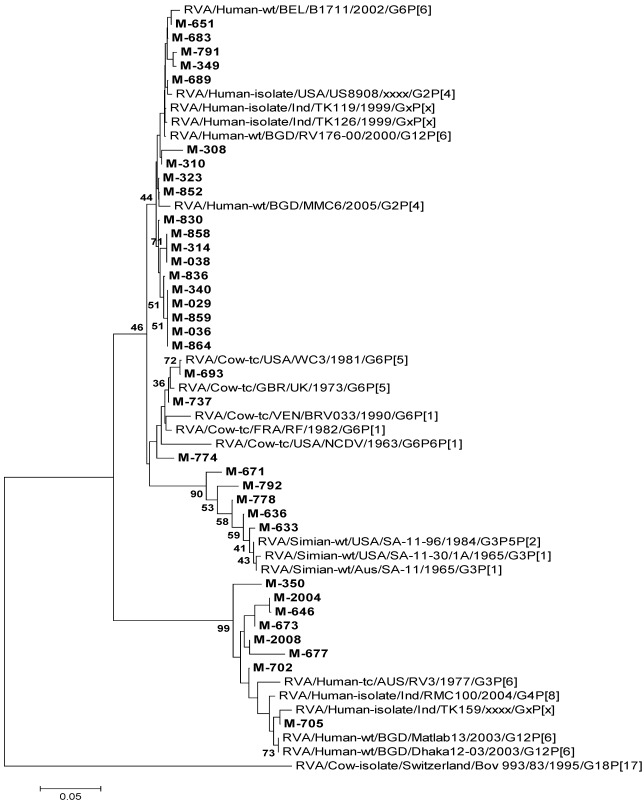
Phylogenetic tree based on the partial nucleotide sequences of VP6 gene (174 bp) of RVA strains. The strains of the present study are in bold face. Scale indicates genetic distance.

Of the 35 [nineteen (March) and sixteen (October)] RVA strains sequenced for VP6 gene (220 bp), 19 [54.3%, four (March) and fifteen (October)] and 8 [22.8%, seven (March) and one (October) showed 91.5–100% nucleotide identities with reference human RVA strains. Five (14.2%) and 3 (8.5%) strains, all from March, were closer to simian and bovine strains of RVAs, respectively, with 89.4–100% nucleotide identities (Figure 2).

Eleven (31.4%, all from October) and 20 [57.1%, ten (March) and ten (October)] of 35 strains were typed for VP7 (G) and VP4 (P) genes, respectively, while 10 (28.6%) all from October were typed for both genes. Distribution of genotypes of RVAs detected in the cases of gastroenteritis from March and October is shown in Table 3.

#### 3.4.2. RVB

**Figure 3:**
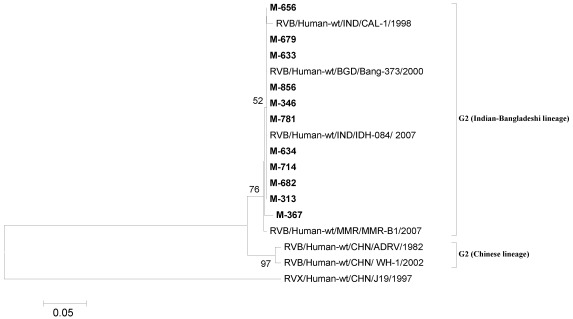
Phylogenetic tree based on the partial nucleotide sequences of NSP2 gene (150 bp) of RVB strains. The strains of the present study are in bold face. Scale indicates genetic distance.

Phylogenetic analysis of NSP2 gene (229 bp) sequences of all 11[seven (March) and four (October)] RVB strains showed clustering with the strains from Indian-Bangladeshi lineage of genotype G2 (Figure 3, Table 3). The nucleotide identity with the Indian, Bangladeshi and Myanmarese strains was noted to be 99.1–100% while it was 92.9–94.7% with Chinese strains (ADRV and WH-1).

**Table 3:** Distribution of genotypes of enteric viruses detected in the outbreak cases of gastroenteritis.

Enteric Virus	Genotypes	March 2006	October 2006
RVA (*n* = 35)	G1P[8]	0	2
	G2P[4]	0	6
	G9P[4]	0	1
	G12P[4]	0	1
	Only G typed	0	11
	Only P typed	10	10
	Both G and P nontypeable	9	5
RVB (*n* = 11)	G2	7	4
NoV (*n* = 10)	GII.2	2	0
	GII.4	3	2
	GII.7	1	0
	GII.12	0	1
	GII.3 + GII.13	0	1
AdV (*n* = 16)	Type 12	2	0
	Type 31	0	1
	Type 40	7	6
HAstV (*n* = 3)	HAstV-7	0	1
	HAstV-8	1	1
EV (*n* = 19)	EV-76	1	3
	EV-84	0	1
	EV-89	0	1
	EV-90	1	3
	CA-13	1	0
	CA-17	0	1
	CA-19	1	0
	CA-21	2	0
	CA-19/22	1	0
	Echo-21	0	1
	Echo-32	0	2
AiV(n = 4)	Genotype B	1	3

#### 3.4.3. NoV

**Figure 4:**
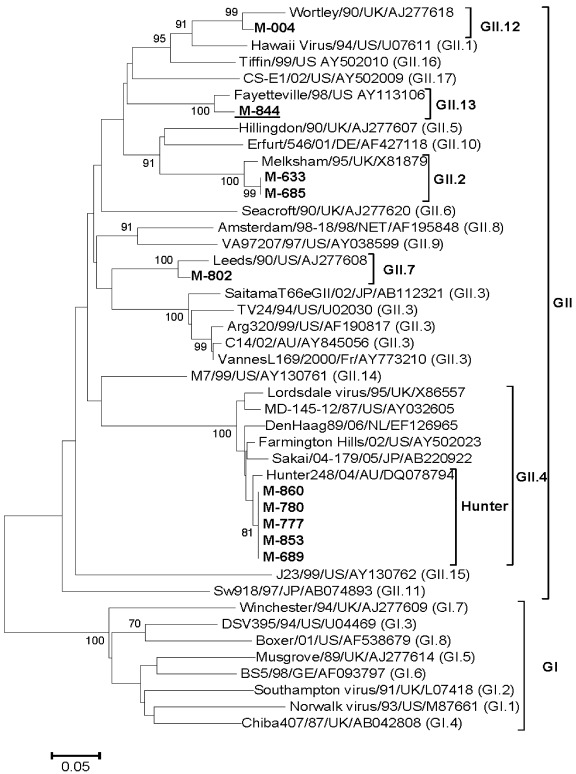
Phylogenetic tree based on the partial nucleotide sequences of VP1 gene (300 bp) of NoV strains. The strains of the present study are in bold face and the strain indicating recombination is underlined. Scale indicates genetic distance.

All of the 16 [nine (March) and seven (October)] NoV strains sequenced for RdRp gene (126 bp) showed the presence of NoV GII and absence of NoV GI. Ten [62.5%, six (March) and four (October)] of the 16 strains were genotyped (Table 3). Phylogenetically, five (50%) strains clustered in Hunter sub-cluster of GII.4 indicating 98.0–98.5% nucleotide identities with the Hunter 248/04/AU (DQ078794) strain. Two (20%), one (10%) and one (10%) strains showed GII.2, GII.7 and GII.12 specificities and presented 97%, 95.8% and 94.6% nucleotide identities, respectively, with the reference strains Melksham/95/UK (X81879), Leeds/90/US (AJ277608) and Wortley/90/UK (AJ277618) (Figure 4). The remaining one (10%) strain, M844, showed the presence of recombination with GII.3 and GII.13 specificities of RdRP and capsid genes, respectively in contiguous sequence. This strain showed 86.9% and 97% nucleotide identity with the reference strains TV24/94/US (U02030, GII.3) and Fayetteville/98/US (AY113106, GII.13) in RdRP and capsid regions, respectively. 

#### 3.4.4. AdV

**Figure 5:**
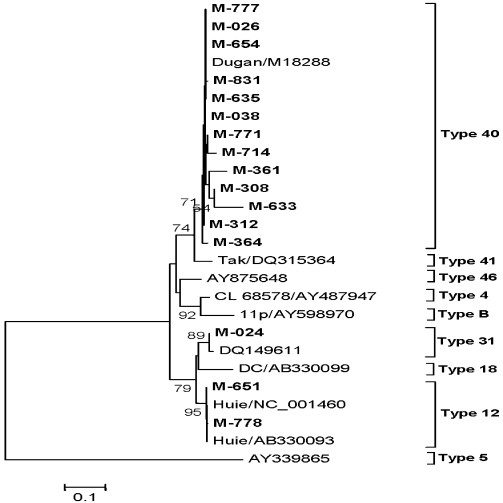
Phylogenetic tree based on the partial nucleotide sequences of Hexon gene (250 bp) of AdV strains. The strains of the present study are in bold face. Scale indicates genetic distance.

Sequence analysis of hexon gene (300 bp) from 16 [nine (March) and seven (October)] AdV strains revealed clustering of 13 [81.2%, seven (March) and six (October)] with the AdV type 40, Dugan strain (M18288, UK), two (12.5%, all from March) with the AdV type 12 Huie strain (AB330093, Japan) and one (6.3% from October) with Austrian AdV type 31 strain (DQ149611) with 97.3–100% nucleotide identities (Figure 5, Table 3).

#### 3.4.5. HAstV

**Figure 6:**
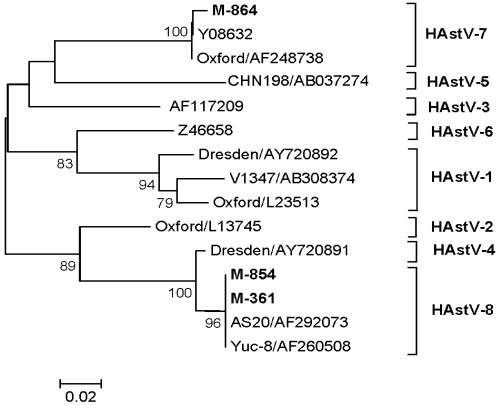
Phylogenetic tree based on the partial nucleotide sequences of ORF2 (190 bp) of HAstV strains. The strains of the present study are in bold face. Scale indicates genetic distance.

Phylogenetic analysis of ORF2 (232 bp) sequences of three HAstV strains showed clustering of two, one each from March and October, with the type 8 strain Yuc-8 (AF260508, Mexico) and that of one (October) with the type 7 strain (Y08632, Norway) with 100% and 99.4% nucleotide identity, respectively (Figure 6, Table 3). 

#### 3.4.6. EV

**Figure 7:**
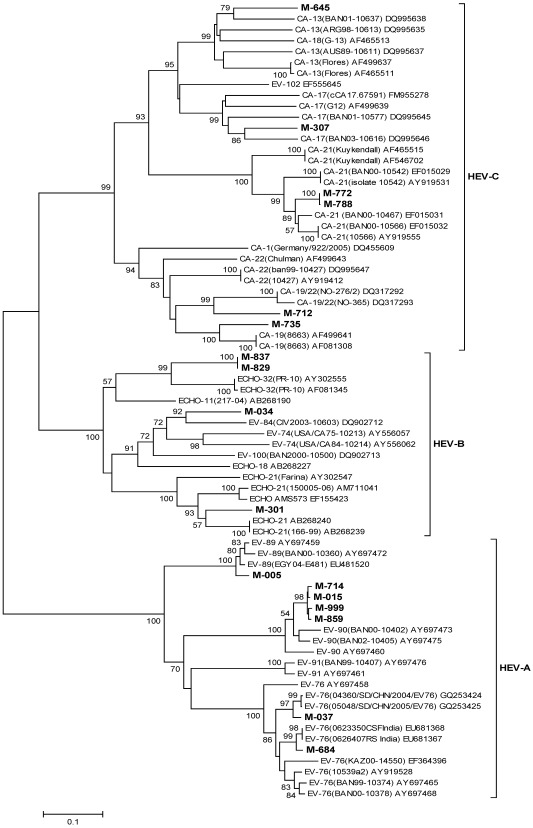
Phylogenetic tree based on the partial nucleotide sequences of VP1/2A gene (363 bp) of EV strains. The strains of the present study are in bold face. Scale indicates genetic distance. * Human Enterovirus.

Of the 36 [sixteen (March) and twenty (October)] EV strains amplified for 5′ NCR, only 19 [52.8%, seven (March) and twelve (October)] were genotyped (Table 3). Phylogenetic analysis of the sequences obtained on the basis of VP1/2A region (450 bp) for 17/19 strains revealed the presence of EV-76 in two, EV-84 in one, EV-89 in one, EV-90 in four, CA-13 in one, CA-17 in one, CA-19 in one, CA-19/22 in one, CA-21 in two, Echo-21 in one and Echo-32 in two strains and indicated 81.4–98.0% nucleotide identities with the corresponding reference strains (Figure 7). The remaining two strains were classified as EV-76 on the basis of VP1 gene sequences (data not shown).

#### 3.4.7. AiV

**Figure 8:**
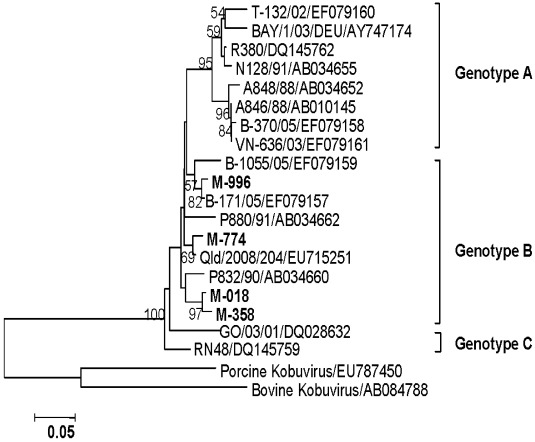
Phylogenetic tree based on the partial nucleotide sequences of 3C-3D junction region (185 bp) of AiV strains. The strains of the present study are in bold face. Scale indicates genetic distance.

Phylogenetic analysis of the sequences obtained for 3C-3D junction region (265 bp) classified all of the 4 [one (March) and three (October)] AiV strains in genotype B and showed 93.7–98.9% nucleotide identity with B-171/05 (EF079157) and QId/2008/204 (EU715251) strains from Bangladesh and Australia, respectively (Figure 8, Table 3 ).

### 3.5. Environmental Investigation

The inspection of the localities affected by the outbreaks of gastroenteritis indicated the existence of densely populated slums with poor hygienic practices and sanitary conditions. The toilet facilities were shared by the residents of the localities. These localities were supplied with water twice daily through age-old pipes by Municipal Corporation. Examination of drinking water and drainage systems of the affected areas revealed the occurrence of both on the same route and leakages in the pipes supplying drinking water and in those of drainage. However, the water samples collected in March, 2006 from two different sites were tested negative for seven different enteric viruses.

## 4. Discussion

To date, the occurrence of more than one enteric virus in gastroenteritis outbreaks has been reported from many countries [33,34]. However, only a single viral agent associated gastroenteritis outbreaks have been reported from India [24,35,36,37]. Most of these outbreaks except a few remained to be characterized for the genotypic specificities of causative viral pathogens. The present study describes detection of the multiple enteric viral infections in the two outbreaks of acute gastroenteritis occurred in southern Mumbai in 2006. A marked increase in the occurrence of cases of gastroenteritis in about a 2 week period each from March and October, 2006 was revealed during the examination of record of all of the months of 2006 and the previous year from both hospitals (Figure 1). The number of cases declined during next two months and remained at usual frequency of admission for gastroenteritis. The records also underscored the presence of disease in patients of all age groups in both outbreaks.

Interestingly, both outbreaks that occurred in different seasons showed predominance of RVA and EV infections. However, frequencies of the occurrence of these viruses and also of other enteric viruses were not different in two incidents (Table 1, *p* > 0.05). Commonly circulating RVA genotypes have been found to be associated with the outbreaks of gastroenteritis that occurred worldwide [38,39,40]. In the present study, RVA strains of G1P[8] and G2P[4] specificity that have been described earlier in sporadic infections in Mumbai were detected only in October, while the strains from the March outbreak remained nontypeable [16]. The other enteric viruses displayed similar genotypic specificities in the two outbreaks. 

The higher prevalence of NoV GII over GI in acute gastroenteritis cases has been reported from India and other parts of the World [15,41]. The NoV genotypes detected in the Mumbai outbreaks also belonged to GII (GII.4, GII.7, GII.12 and GII.3/GII.13). This finding was similar to that reported earlier for the sporadic infections from Western India [15]. It is of note that GII.2 identified only in Mumbai was not detected in any other city from Western India, while GII.b infections were detected in cities other than Mumbai [15]. In view of this observation, a separate molecular epidemiological study is required to know the diversity of NoVs circulating in Mumbai. 

In the present study, EV-76, EV-84, EV-89, EV-90, CA-13, CA-17, CA-19, CA-19/22, CA-21, Echo-21 and Echo-32 types were identified for the first time in Western India. However, Echo-11 identified in the gastroenteritis outbreak from Southern India [35] was not detected. The high prevalence and genetic diversity of EV strains noted in the present study are indicative of the association of EVs with acute gastroenteritis and suggest the need for surveillance of EV infections in India. AiVs have been described to be present in the mixed infections together with other enteric pathogens [40]. Interestingly, in this study, all four patients excreting AiV showed absence of other enteric viruses.

RVA, AdV, calicivirus, HAstV and other viruses have been described to be excreted continually in patients with immunodeficiency syndrome [42]. However, such a syndrome was not recorded in an infant who showed presence of infection with RVA, RVB, NoV and Adv in this study. 

The present study identified single and mixed enteric viral infections during the outbreaks of gastroenteritis and suggested that the affected individuals were highly exposed to multiple enteric viruses. The study documented for the first time the circulation of RVB, AdV, HAstV and AiV in Mumbai. Most of the enteric viruses examined in the study were detected in both summer (March) and winter (October) months. These findings indicate the necessity of monitoring of sporadic cases of gastroenteritis in Mumbai city for single and mixed enteric viral infections. 

Viruses causing acute gastroenteritis are known to be excreted in large quantities in human faeces and find their way out in the sewage system. Thus, sewage water is a good source of a variety of enteric viruses circulating in any region/community [40]. An accidental mixing of the sewage water with the drinking water supply provides an opportunity to multiple enteric viruses to infect humans simultaneously and cause disease outbreaks. In the present study, water samples tested negative for enteric viruses. However, contamination of drinking water was reflected by brown color and malodor noticed by the inhabitants prior to the occurrence of outbreak, thus indicating leakages in the pipelines. The information available from the affected localities indicated that the water supplying pipes were more than 100 years old and that unauthorized and unclaimed pipelines existed for water connection. Since the daily water supply in this area was intermittent, back siphoning of sewage water in the drinking water supplying pipes could not be ruled out. Subsequently, clusters of cases who developed diarrhea and/or vomiting were reported from a single locality in a short period representing rampant infections of gastroenteritis [43,44]. These findings highlight the possible enhancing impact of the sewage contamination on the endemic transmission of prevalent and emerging strains of enteric viruses. Notably, majority of the viral agents (RVA, NoV, AdV, HAstV and EV) detected in the patients affected by outbreaks showed the presence of more than one genotype/serotype indicating diverse nature of enteric viral pathogens. Thus, this report provides strong evidence of the cocirculation of multiple enteric viruses in Mumbai, the biggest cosmopolitan city of India. To the best of our knowledge, this is the first report from India that documents coexistence of seven (RVA, RVB, NoV, AdV, HAstV, EV and AiV) important enteric viruses in the outbreak cases of acute gastroenteritis and emphasizes the need to investigate such outbreak cases in different parts of India to evaluate the health risk associated with enteric viral pathogens in Indian population.

## 5. Conclusions

This investigation reports association of seven different enteric viruses in two outbreaks of gastroenteritis in Mumbai, India, one of the most densely populated cities in the World. Virological examination of the faecal specimens from the outbreaks ascertained the predominance of RVAs and EVs in the study population. Therefore, intervention strategies need to be developed against these viral infections. Occurrence of two outbreaks within seven months period in the same region emphasizes the need for constant vigilance of water supply and drainage systems to reduce the risks of such incidences. Overall, the study ratifies the need for continuous surveillance of outbreaks of gastroenteritis in Mumbai and also in other parts of India for identifying the prevalent and new enteric viruses.
